# Genetic screening of PLA1/PLA2 polymorphous marker of integrin beta 3 (ITGB3) GP IIIA gene in adolescent girls with puberty menorrhagia

**DOI:** 10.25122/jml-2022-0350

**Published:** 2023-02

**Authors:** Yuliia Vasylivna Tsysar, Oksana Anatolievna Andriiets, Liudmyla Vasylivna Dubyk, Kristina Viktorivna Dyak, Raluca Mihaela Radu

**Affiliations:** 1 Department of Obstetrics and Gynecology, Bukovinian State Medical University, Chernivtsi, Ukraine; 2 Materno-Fetal Assistance Excellence Unit, Polizu Clinical Hospital, Alessandrescu-Rusescu National Institute for Mother and Child Health, Bucharest, Romania

**Keywords:** adolescent girls, menorrhagia, thyroid gland, ITGB3 (GP IIIA) gene polymorphism

## Abstract

Puberty menorrhagia is one of the urgent problems of modern reproductive medicine. The study aimed to investigate the relationship between polymorphism of the GP IIIa (PLA1/PLA2) gene and improve the diagnosis of puberty menorrhagia in girls with thyroid gland pathology. Ninety-seven girls at puberty age were divided into three groups: group 1 (main) – girls with puberty menorrhagia and thyroid gland pathology (30 individuals), group 2 (comparison) – 40 girls with puberty menorrhagia, group 3 (control) – 27 practically healthy girls. Polymorphism of the GP IIIa (PLA1/PLA2) gene was studied by isolating genomic DNA from peripheral blood leukocytes, followed by amplification with a polymerase chain reaction. Results showed that mutation in the 17^th^ chromosome of q21.32 of the GP IIIa gene occurred in 8.6% of cases among adolescents with menorrhagia, in contrast to the control group, where it was not observed at all. The A1A1-genotype occurred by 11.7% (X^2^=4.01, p=0.041) more often in adolescents with menorrhagia than in girls with concomitant thyroid gland pathology and by 15.0% (X^2^=4.54, p=0.033) more often than in the control group. It was also found that the presence of the A1A2-genotype unreliably reduced the chances of uterine bleeding in adolescent girls by 1.45 times (OR=2.12) and was a protective factor in the puberty menorrhagia occurrence (OR=0.47). It may be concluded that the identification of a hereditary factor of the reproductive system diseases of adolescent girls fundamentally changes the point of view on the tactics of disease management and subsequent therapy.

## INTRODUCTION

The current generation is characterized by a significant increase in adolescent girls with puberty menorrhagia, a severe form of menstrual dysfunction that often occurs with concomitant endocrine system disorders [1]. It is worth noting that thyroid gland pathology plays a significant role in the structure of the endocrine factor of puberty menorrhagia [2].

One way to better understand the causes and development of menstrual dysfunction in teenage girls is to use genetic research methods. This approach has become increasingly important in clinical practice in recent years. Establishing a hereditary genesis in the diseases of the female reproductive system can fundamentally change how the disease is managed and treated [3, 4].

According to a multifactorial type of heredity, the inherited predisposition for menstrual dysfunctions considers that genetic factors (the total effect of several genes) appear only when exposed to additional adverse environmental factors. These patients often have a history of a high infectious index, stressful situations, occupational hazards, and inadequate environmental factors that can destabilize the genome and activate pathological genes [5].

Sexual differentiation and the normal function of the reproductive system are directly dependent on the morphological and functional state of sex chromosomes and genes. This has been proven by various variants of violations of sexual differentiation associated with anomalies in the genes responsible for menstrual function formation [6]. Studies have shown that a number of alleles of certain genes are involved in the regulation of the reproductive system. The development of new methods for differentiating gene polymorphisms has significantly increased the ability of cytogenetic analysis. It became possible to determine the configuration of gene alleles, which makes it possible to reveal the prerequisites for certain dysplastic configurations that can occur even during puberty [7].

The division of women with allelic conformity according to the GP IIIa gene and the content of embryo-specific antibodies makes it possible to predict isolated forms of hyperplastic processes with high probability. For example, the PLAI allele of the GP IIIa gene carriership determines the genetic predisposition to the occurrence of endometrium hyperplastic processes and bleeding development, and the PLAII allele of the GP IIIa gene carriership excludes the occurrence of adenomyosis and uterine fibroids. Decreasing the frequency of PLAI of the GP IIIa gene occurrence by 1.5-2 times in hyperplastic processes suggests that there are genetic factors that determine the development of this pathology [7]. Studying the genes that encode different families of integrin receptors and their relationship with the development of hyperplastic processes in the reproductive organs is particularly promising in identifying the key mechanisms behind menstrual disorders in adolescent girls. Advanced cytogenetic diagnostic methods in examining adolescent girls with metrorrhagia can help identify a risk group based on the allelic conformity of the GP IIIa gene [8].

It is recommended to conduct a study that aims to determine allelic affiliations for the GP IIIa gene, an integral genetic factor that reflects the state of fine intercellular interactions to identify groups at risk for the development of endometrium hyperplastic processes in girls, as well as early detection of pathological proliferative transformation of the endometrium [9].

The purpose of this study was to improve the diagnostic method for puberty menorrhagia in thyroid gland pathology by identifying the GP IIIa gene (PLA1/PLA2) polymorphism.

## MATERIAL AND METHODS

This study was conducted in 2011–2015 and included the clinical and laboratory examination of 97 adolescent girls, who were divided into three groups: group 1 (main) – adolescent girls with menstrual irregularities (puberty menorrhagia) with concomitant thyroid pathology (30 persons), group 2 (comparison) – 40 adolescent girls with menstrual disorders in the form of puberty menorrhagia, group 3 (control) – 27 practically healthy puberty age girls Examination of external genitalia was performed in the presence of parents, relatives or caretakers (parents/caretakers).

The inclusion criteria for this study were:

- Age of the participant between the onset of menarche and 18 years old;
- Presence of menstrual irregularities at the time of examination;
- Absence of any known somatic pathology;
- Presence of concomitant thyroid pathology.

The exclusion criteria from the study were:

- Age over 18 years;
- Absence of menarche;
- Secondary amenorrhea;
- Presence of an infectious factor;
- Concomitant extragenital pathology (except for thyroid pathology);
- Diagnosis of hyperprolactinemia;
- Girls with Stein-Leventhal syndrome.

The GP IIIa (PLA1/PLA2) gene polymorphism was studied once, after the inclusion of patients in the study, by isolating genomic DNA from peripheral blood leukocytes, followed by amplification of the polymorphous region using polymerase chain reaction (PCR) in Amply-4l thermal cycler, with individual temperature for primer of the corresponding gene. Individuals homozygous for the insertion allele of the GP IIIa gene were tested using the additional pair of primers located on the long arm of the corresponding chromosome. The DNA extraction was carried out using DNA-Sorb-B reagents according to the instructions. Purified DNA was stored at -20±2℃. Samples for PCR analysis were prepared for each patient using the AmpliSen-200-1 kit. The amplification was made according to the individual temperature regime of primer attachment to single-strand DNA chains of the gene. To discriminate alleles of the GP IIIa (PLA1/PLA2) gene, restriction endonuclease from Fermentas® (Lithuania) was used. Fragments were visualized using an ultraviolet-light source in the presence of 100–1000 bp SibEnzyme molecular weight markers.

Statistical processing of the material was carried out using Statistica and Microsoft Excel Windows computer programs from StatSoft^®^ Inc.

## RESULTS

An in-depth analysis of menstrual irregularities was conducted among 70 adolescent girls divided into groups: group 1 (main) – 30 girls with menstrual irregularities caused by thyroid gland pathology and group 2 (comparison) – 40 adolescent girls with menstrual cycle disorders in the form of puberty menorrhagia. All patients were examined and treated at the Chernivtsi Regional Perinatal Center during 2011–2015. Additionally, 27 adolescent girls (aged 12–17) with a physiological puberty period, who made up the control group, were examined at the Chernivtsi City Children's Clinic.

The study was conducted using a standardized protocol which included: a passport section that recorded the age, social engagement, somatic and gynecological anamnesis, as well as the characteristics of the pre-puberty and puberty periods. All participants had menstrual irregularities in the form of puberty menorrhagia or hyperpolymenorrhea.

We studied genetic and molecular predictors associated with puberty menorrhagia caused by the activity of receptors for glycosylated platelet glycoprotein GP IIIa (integrin beta 3 – ITGB3). Since the Leu33Pro polymorphism of the GP IIIa gene may be one of the main causes of genetically determined dysregulation of the hemostasis system and sensitivity to hemostatic therapy, the frequency of alleles and A1A2-genotypes of the polymorphism of the GP IIIa gene was determined in adolescents with menorrhagia, including the background of thyroid gland pathology and in healthy girls.

The distribution of alleles for the polymorphic locus of the ITGB3 (GP IIIa) gene among the participants generally corresponds to the expected Hardy-Weinberg equilibrium (Table 1). There was no statistically significant difference between the expected and actual heterozygosis in patients in the main group, with its probable inbreeding coefficient (F) in the control group (F=-0.43, X^2^=5.78, p=0.016). In quantitative terms, the dominant allele was the A1 variant (70.5%), which accordingly affects the allele balance, provoking the unreliable population surplus of heterozygosis, but does not significantly violate the overall population distribution in the sample (F=-0.11, X^2^=2.28, p>0.05).

**Table 1:** Analysis of heterozygosis and allelic state of A1/A2 polymorphism of the ITGB3 (GP IIIa) gene.

Groups	Alleles, n (%)		P_A1_	P_A2_	H_0_	H_E_	F	X^2^	P
	A1	A2							
**Survey group, n=140**	99 (70.7)	41 (29.3)	0.71	0.29	0.41	0.41	-0.00	<1.0	>0.05
**Control group, n=50**	35 (70.0)	15 (30.0)	0.70	0.30	0.60	0.42	-0.43	5.78	0.016
**Total, n=190**	134 (70.5)	56 (29.5)	0.71	0.29	0.46	0.41	-0.11	2.28	>0.05

1. P_A1_ – the relative frequency of the A1 allele; P_A2_ – the relative frequency of the A2 allele. 2. H_0_ – anticipated heterozygosis; H_E_ – expected heterozygosis; F – the coefficient of inbreeding (relative deviation of genotype frequencies from panmixia) of heterozygous deficiency or excess. 3. X^2^ & p – the validity criterion of the "null" hypothesis between actual and expected heterozygosis. 4. n (%) – the number (percentage) of observations.

The distribution of genotypes of the polymorphic locus of the ITGB3 (GP IIIa) gene corresponded to the expected Hardy-Weinberg population equilibrium (Table 2), both in general and separately in the examined group. The A1 allele dominated quantitatively in the examined group, especially in participants with menorrhagia without lesions of the thyroid gland by 2.85 times (74.0% *versus* 26.0% of those with the A2 allele), slightly less in those with concomitant thyroid gland pathology by 2.03 times (67.0% *versus* 33.0%). However, this did not disturb the allele balance in the sample of patients and, in general, and compensated for the heterozygosity deficiency (F=0.03, p>0.05) with its excess (F=-0.05, r>0.05).

**Table 2:** Analysis of heterozygosis and allelic state of the ITGB3 (GP IIIa) gene considering the burden of puberty menorrhagia with thyroid gland pathology.

Groups, n	Genotypes, n (%)			P_A1_	P_A2_	H_E_	H_0_	F	X^2^	P
	A1A1	A1A2	A2A2							
**1**	2	3	4	5	6	7	8	9	10	11
**Puberty menorrhagia, n=40**	22 (55.0)	15 (37.5)	3 (7.5)	0.74	0.26	0.38	0.39	0.03	1.03	>0.05
**Puberty menorrhagia + thyroid gland pathology, n=30**	13 (43.3)	14 (46.7)	3 (10.0)	0.67	0.33	0.47	0.44	-0.05	1.05	>0.05
**Total patients, n=70**	35 (50.0)	29 (41.4)	6 (8.6)	0.71	0.29	0.41	0.41	-0.00	<1.0	>0.05
**Control group, n=25**	10 (40.0)	15 (60.0)	0	0.70	0.30	0.60	0.42	-0.43	5.78	0.016
**Total, n=95 (%)**	45 (47.4)	44 (46.8)	6 (6.3)	0.71	0.29	0.46	0.41	-0.11	2.28	>0.05

1. P_A1_ – the relative frequency of the A1 allele; P_A2_ – the relative frequency of the A2 allele. 2. H_0_ – anticipated heterozygosis; H_E_ – expected heterozygosis; F – the coefficient of inbreeding (relative deviation of genotype frequencies from panmixia) of heterozygous deficiency or excess. 3. X^2^ & p – the validity criterion of the "null" hypothesis between actual and expected heterozygosis. 4. n (%) – the number (percentage) of observations.

We analyzed the increase/decrease in absolute (ARI/ARR) and relative (RRI/RRR) risks, relative risk indicators (RelR), odds ratio (OR), and risks (RR) with the determination of confidence intervals [95% (CI)] to determine potential risk factors for the occurrence of puberty menorrhagia in adolescents, considering the genetic component (Table 3).

**Table 3:** Alleles and genotypes of the A1/A2 polymorphic locus of the ITGB3 (GP IIIa) gene as risk factors for puberty menorrhagia.

No.	Potential risk factor	Puberty menorrhagia in adolescent girls							Absence of menstrual disorders						
		ARI/ARR	RRI/RRR	RelR	RR	OR	95% CI RR/95% CI OR	X^2^ p	ARI/ ARR	RRI/RRR	RelR	RR	OR	95% CI RR/95% CI OR	X^2^ p
**1**	A1A1-genotype	-0.1	-0.25	1.25	1.11	1.5	0.87-1.41/ 0.59-3.79	X^2^<1.0 p>0.05	0.1	0.2	0.8	0.74	0.67	0.37-1.48/ 1.21-10.39	X^2^<1.0 p>0.05
**2**	A1A2-genotype	0.19	0.31	0.69	0.82	0.47	0.64-1.05/ 0.19-1.20	X^2^=1.86 p>0.05	-0.19	-0.45	1.45	1.74	2.12	0.87-3.47/ 0.84-5.38	X^2^=2.56 p>0.05
**3**	A2A2-genotype (hypothetical)	-0.006	-0.07	1.07	1.02	1.08	0.67-1.55/ 0.20-5.72	X^2^<1,0 p>0.05	0.006	0.07	0.93	0.95	0.93	0.27-3.30/ 0.17-4.93	X^2^<1.0 p>0.05
**4**	A1 allele	-0.01	-0.01	1.01	1.01	1.03	0.84-1.22/ 0.51-2.10	X^2^<1.0 p>0.05	0.007	0.01	0.99	0.98	0.97	0.58-1.64/ 0.48-1.96	X^2^<1.0 p>0.05
**5**	A2 allele	0.007	0.02	0.98	0.99	0.97	0.82-1.19/ 0.48-1.96	X^2^<1.0 p>0.05	-0.01	-0.02	1.02	1.02	1.03	0.61-1.72/ 0.51-2.09	X^2^<1.0 p>0.05

1. ARI (absolute risk increase)/ARR (absolute risk reduction) – absolute risk increase/reduction; 2. RRI (relative risk increase)/RRR (relative risk reduction) – relative risk increase/reduction; 3. RelR (relative risk) – relative risk; 4. RR (Risk Ratio) – risk ratio; 5. OR (Odds Ratio) – odds ratio; 6. 95%CI RR, OR (confidence interval) – Confidence intervals for risk ratio (RR), odds (OR).

A risk factor was considered clinically significant when the odds ratio was greater than 1.20. The results showed that the presence of the A1A1-genotype of the ITGB3 (GP IIIa) gene in adolescents greatly increases the relative risk of menorrhagia by 1.25 times (OR=1.50, p>0.05). The presence of the A1A2-genotype unreliably reduces the chances of menstrual disorders by 1.45 times (OR=2.12, p>0.05) and is a protective factor in the occurrence of menorrhagia (OR=0.47, p>0.05) (Table 3). The allelic state of the GP IIIa gene does not affect the risk of puberty menorrhagia or the lack thereof (p>0.05).

## DISCUSSION

There are many known hereditary factors, including genetic ones, that can indirectly cause disorders in thrombocytic-vascular hemostasis or trigger disorders in fibrinolysis [10].

Several polymorphisms in genes of the hemostasis system are of particular interest, such as the mutation of factor V, factor II, mutation of the gene for the PAI-1 plasminogen activator inhibitor of the fibrinogen β-chain [11], polymorphism of the thrombocytic GP IIIa fibrinogen receptor, integrin α2, platelet glycoprotein 1B mutation [12], polymorphism of methylene-tetrahydrofolate reductase and methionine synthase reductase, which are associated with the homocysteine metabolic disorder. A low homocysteine concentration can cause bleeding, while high ones can cause thrombosis [13]. There is active discussion and ongoing scientific research on the genetically determined participation of procoagulants in fibrin- and thrombogenesis– factors VIII, von Willebrand, fibrinogen ones etc [14–20]. The important role among the factors that determine the activity of the anticoagulant or procoagulant potential and fibrinolysis in the pathogenesis of early puberty menorrhagia is also played by background somatic (puberty hypertension) and infectious diseases (bacterial toxins, viral infection, IL-TN), the tumor process, systemic inflammatory diseases of the connective tissues (oxidized lipoproteins, immune complexes), hemolytic anemia, hyperhomocysteinemia, dyslipidemia, mesenchymal dysplasia, antiphospholipid syndrome, chronic stress, hormonal imbalance etc [21–24].

The appearance of early menorrhagia in the availability of the genetic "favorable" and background initiating factor leads to endothelium dysfunction and damage to the vessel wall, followed by the release of von Willebrand factor and P-selectin into the blood, the latter triggering a cascade of the hemostasis system: recognition of damage to the endothelial wall, adhesion and aggregation of blood platelets. G-protein-coupled receptors activate heterodimeric GP IIb-IIIa platelet receptors, and this complex undergoes conformational changes through Ca^2+^-dependent mechanisms, which ensures the binding of the platelet itself to fibrinogen [24, 25]. There are dysregulation problems and coagulation – bleeding, or thrombosis at the dysfunction of the third stage of hemostasis (platelet aggregation) at the level of platelet glycoprotein receptors GP IIIa (decrease in the amount and activity of this glycoprotein), which are the only ones that provide the above-described connection of the platelet with fibrinogen [26].

Glycoprotein platelet fibrinogen receptors play a key role in platelet adhesion and aggregation during blood clot formation, which allows considering them as "candidate genes" for studies through association with acute vascular events in the pathology of internal organs: spontaneous abortions, miscarriage, puberty menorrhagia, acute coronary syndrome, cerebral strokes etc [27–30].

Today, 18 mutations of the GPIIIa gene are known, found in 8 variants in the European population that differ in the amino acid sequence in 6 positions. Only a point mutation at position 33 of the GP IIIa protein that leads to the replacement of leucine (Leu) by proline (Pro), which is the result of transversion in the exon of the GP IIIa gene at position 1565, is of clinical interest. The results of studies on the functional activity of the A1A2 polymorphism of this gene are contradictory and vary significantly across populations. According to some authors, platelets carrying GPIIIa with proline at position 33 have a lower activation threshold and are also more sensitive to the effects of myocardial infarction and breast and ovarian cancer [31–34].

Thus, the issue of the relationship between the A1A2 polymorphic locus of the GP IIIa gene and the development of thrombo-hemorrhagic complications remains open and requires further exploration.

## CONCLUSION

The A1A1-genotype is more common in adolescent girls with menorrhagia without thyroid gland pathology than in girls with menorrhagia and thyroid gland disease. The relative frequency of the A1A2-genotype and A2A2-genotype in girls with menorrhagia and pathology of the thyroid gland marginally predominates over those in adolescent girls in the comparison group without thyroid gland problems. Carriers of the A1A1-genotype are more common among girls with puberty menorrhagia than in the control group, and there are more heterozygous carriers of the A1A2-genotype in the control group than in both examined groups.

## Personal thanks

The authors thank all participants of this study.
